# Motor neuron and pancreas homeobox 1/HLXB9 promotes sustained proliferation in bladder cancer by upregulating CCNE1/2

**DOI:** 10.1186/s13046-018-0829-9

**Published:** 2018-07-16

**Authors:** Mingkun Chen, Rongpei Wu, Gang Li, Cundong Liu, Lei Tan, Kanghua Xiao, Yunlin Ye, Zike Qin

**Affiliations:** 1 Department of Urology, The Third Affiliated Hospital of Southern Medical University, Guangzhou, 510630 Guangdong China; 2 Department of Urology, the First Affiliated Hospital, Sun Yat-sen University, Guangzhou, 510080 Guangdong China; 3 Department of Urology, Guangzhou Red Cross Hospital, The Affiliated Hospital of Medical College of Ji-Nan University, Guangzhou, 510220 Guangdong China; 4 Department of Urology, Sun Yat-sen University Cancer Center, State Key Laboratory of Oncology in South China, Collaborative Innovation Center for Cancer Medicine, Guangzhou, 510060 Guangdong China; 5 Department of Urology, Cancer Center, Sun Yat-sen University, Guangzhou, Guangdong 510060 China

**Keywords:** Bladder cancer, Proliferation, MNX1, CCNE1, CCNE2

## Abstract

**Background:**

Uncontrolled proliferation is thought to be the most fundamental characteristic of cancer. Detailed knowledge of cancer cell proliferation mechanisms would not only benefit understanding of cancer progression, but may also provide new clues for developing novel therapeutic strategies.

**Methods:**

In vitro function of MNX1 (Motor neuron and pancreas homeobox 1) in bladder cancer cell was evaluated using MTT assay, colony formation assay, and bromodeoxyuridine incorporation assay. Real-time PCR and western blotting were performed to detect MNX1 and CCNE1/2 expressions. In vivo tumor growth was conducted in BALB/c-nu mice.

**Results:**

We reported that MNX1 is responsible for sustaining bladder cancer cell proliferation. Abnormal MNX1 upregulation in bladder cancer cell lines and 167 human tissue specimens; high MNX1 expression levels correlated significantly with shorter 5-year overall and relapse-free survival in the bladder cancer patients. Furthermore, MNX1 overexpression accelerated bladder cancer cell proliferation and tumorigenicity both in vitro and in vivo, whereas MNX1 downregulation arrested it. In addition, MNX1 transcriptionally upregulated CCNE1 and CCNE2 by directly bounding to their promoters, which promoted G1–S transition in the bladder cancer cells.

**Conclusion:**

These findings reveal an oncogenic role and novel regulatory mechanism of MNX1 in bladder cancer progression and suggest that MNX1 is a potential prognostic biomarker and therapeutic target.

**Electronic supplementary material:**

The online version of this article (10.1186/s13046-018-0829-9) contains supplementary material, which is available to authorized users.

## Background

Bladder cancer is a primary cause of morbidity and mortality, with 429,700 estimated new cases each year and 165,000 deaths worldwide [1]. Treatment for bladder cancer has improved greatly in recent decades, however, many patients especially those at the advanced stages of the disease, succumb to it. Although localized bladder cancer can be excised by surgery, the recurrence and progression rates remain high. Patients with advanced bladder cancer mostly receive radiotherapy or chemotherapy, but the therapeutic outcomes remain unsatisfactory [2]. Therefore, there is an urgent need to determine the underlying molecular mechanisms of bladder cancer tumorigenesis and to develop effective, molecular targeted bladder cancer therapies.

The vital feature of cancer is thought to be sustained proliferation [3]. Generally, dysregulation in cell cycle progression results in the release of proliferative signals in cancer cells, consequently disrupting cell number homeostasis and causing uncontrolled cell proliferation. The prevailing model during cell cycle progression is that cyclin-dependent kinase (CDK) activity increases during G1–S transition, which involves the formation of a cyclin–CDK complex. Subsequently, phosphorylation of the retinoblastoma (Rb) family members leads to the release of E2F-containing repressor complexes from E2F-regulated promoters and upregulates the expression of E2F downstream target genes [4]. In detail, CCNE (cyclin E, including CCNE1 and CCNE2) binds to CDK2, and subsequently phosphorylates Rb to promote G1–S progression. It is well-demonstrated that the dysregulation of CCNE–CDK2 activity is involved in many human cancers, including breast, bladder, and lung cancer, resulting in uncontrolled cell proliferation [5–8]. Consistently, CCNE overexpression is associated with poor clinical prognosis of bladder cancer, while inhibiting CCNE–CDK2 activity decreases cell proliferation and tumor formation and is deemed a therapeutic approach in human cancers [9–12]. Therefore, better understanding of how the cell cycle transits would aid comprehension of bladder cancer progression and provide new clues for developing novel therapeutic strategies.

MNX1 (motor neuron and pancreas homeobox 1), also known as HLXB9, is a homeodomain-containing transcription factor [13]. It is located on chromosome 7q36.3 and belongs to the family of EHG homeobox genes that also includes EN1, EN2, GBX1, and GBX2 [14]. Although its normal function is unknown, it is involved in several important pathologies. First, MNX1 mutation or deletion is the primary cause of Currarino syndrome, which is a rare congenital malformation characterized by sacral anomalies, anorectal malformation and presacral mass, suggesting that deregulation of MNX1 may induce abnormal development of tissue and be associated with cell malignant transformation [15, 16]. In addition, MNX1 is a causative oncogene in infant acute myeloid leukemias (AML) [17–20]. Interestingly, several studies have revealed that MNX1 is upregulated in some solid human cancers. Neufing et al. suggested that MNX1 is overexpressed in breast carcinoma compared to the corresponding non-malignant tissue [21]. In agreement with this, Wilkens et al. confirmed HLXB9 upregulation in surgical specimens in a subgroup of poorly differentiated hepatocellular carcinoma [22]. It was also recently reported that MNX1 is a novel oncogene upregulated to a relatively greater degree in prostate cancer [23]. Taken together, MNX1 overexpression may play an important role in tumor development. However, its biological roles and detailed molecular mechanism in human bladder cancer remain unknown.

Herein, we show that MNX1 is obviously upregulated in bladder cancer cells and is associated with poorer prognosis. MNX1 overexpression markedly promoted bladder cancer cell proliferation and tumorigenicity both in vitro and in vivo, whereas MNX1 silencing inhibited it. Furthermore, we found that MNX1 can accelerate G1–S transition in the bladder cancer cell cycle by transcriptionally upregulating CCNE1 and CCNE2 expression. Our results reveal a novel mechanism for the oncogenic role of MNX1 in bladder cancer and suggest MNX1 as a new biomarker and potential therapeutic target.

## Methods

### Human bladder cancer cell lines

SV-HUC-1, T24, MGH-U4, 253 J, 639 V, 5637, RT4, and 575A cells were purchased from American Type Culture Collection and cultured in DMEM with 10% foetal bovine serum (FBS, HyClone, Logan, UT, USA). All cells were incubated at 37 °C in 5% CO_2_ atmosphere.

### Patient information and tissue specimens

This study was conducted on 167 paraffin-embedded, archived bladder cancer samples that had been histopathologically and clinically diagnosed at the Sun Yat-sen University Cancer Center from 2005 to 2011. Additional file 1: Table S1 summarizes the clinicopathological characteristics. Ethical approval from the Institutional Research Ethics Committee and prior patient consent had been obtained for the use of the clinical specimens for research purposes. Freshly collected bladder cancer tissues were frozen and stored in liquid nitrogen until used.

### RNA extraction, reverse transcription and real-time PCR

Total RNA was extracted from cultured cells using the Trizol reagent (Invitrogen, Carlsbad, CA, USA) according to the manufacturer’s instructions. 2 μg extracted RNA from each sample was used for cDNA synthesis with M-MLV Reverse Transcriptase (Promega, Madison, US). cDNAs were amplified and quantified by SYBR-Green in CFX96 Real Time System C1000 Cycler (Bio-Rad Laboratories, Singapore). Expression data were normalized to the housekeeping gene GAPDH and calculated as 2^-[(Ct of *gene*) - (Ct of *GAPDH*)]^, where Ct represents the threshold cycle for each transcript. Primers used in the PCR reactions are listed in the Additional file 2: Table S4.

### Western blotting

Cells were harvested and equal quantities of denatured protein samples were resolved on SDS-polyacrylamide gels, and then transferred onto polyvinylidene difluoride membranes. The membrane was blocked and incubated with primary antibodies, followed by the horseradixh peroxidase-conjugated secondary antibody. Proteins were visualized using ECL reagents. An anti-MNX1 rabbit polyclonal antibody (1:500 dilution; Sigma Aldrich), an anti-CCNE1 Rabbit polyclonal antibody (1:1000 dilution;Proteintech), an anti-CCNE2 Rabbit polyclonal antibody (1:1000 dilution;Proteintech), an anti-α-tubulin mouse monoclonal antibody (1:4000 dilution; Sigma-Aldrich), an anti-Rb rabbit polyclonal antibody (1:1000 dilution; Cell Signaling Technology), an anti-p-Rb Rabbit polyclonal antibody (1:1000 dilution; Cell Signaling Technology), were used in this study.

### Immunohistochemistry (IHC)

Immunohistochemical analysis was done to study altered protein expression in 167 human bladder cancer tissues. In brief, paraffin-embedded specimens were cut into 4-μm sections and baked at 65 °C for 30 min. The sections were deparaffinized with xylenes and rehydrated. Sections were submerged into EDTA antigenic retrieval buffer and microwaved for antigenic retrieval. The sections were treated with 3% hydrogen peroxide in methanol to quench the endogenous peroxidase activity, followed by incubation with 1% bovine serum albumin to block nonspecific binding. Rabbit anti-MNX1 (1:500 dilution; Sigma Aldrich) was incubated with the sections overnight at 4 °C. For negative controls, the rabbit anti-MNX1 antibody was replaced with normal goat serum, or the rabbit anti-MNX1 antibody was blocked with a recombinant MNX1 polypeptide by coincubation at 4 °C overnight preceding the immunohistochemical staining procedure. After washing, the tissue sections were treated with biotinylated anti-rabbit secondary antibody (Zymed), followed by further incubation with streptavidin-horseradish peroxidase complex (Zymed). The tissue sections were immersed in 3-amino-9-ethyl carbazole and counterstained with 10% Mayer’s hematoxylin, dehydrated, and mounted in Crystal Mount.

Two independent pathologists blinded to the clinical outcome scored and evaluated the staining results. The scores were determined by combining the proportion of positively-stained cells and the intensity of staining. Cell proportions were scored as follows: 0, no positive cells; 1, < 10% positive cells; 2, 10–35% positive cells; 3, 35–75% positive cells; 4, > 75% positive cells. Staining intensity was graded according to the following standard: 0, no staining; 1, weak staining (light yellow); 2, moderate staining (yellow brown); 3, strong staining (brown). The staining index (SI) was calculated as the product of the staining intensity score and the proportion of positive cells. Using this method of assessment, we evaluated protein expression by determining the SI, with possible scores of 0, 1, 2, 3, 4, 6, 8, 9, and 12. Samples with a SI ≥ 6 were defined as high expression, and samples with a SI < 6 were defined as low expression. The cutoff value was determined on the basis of a measure of heterogeneity using the log-rank test with respect to 5-year overall and relapse-free survival.

### Plasmids, virus constructs and retroviral infection

The human MNX1 cDNAs were PCR-amplified and cloned into the pMSCV-puro-retro vector (Clontech). Two shRNAs against MNX1 in pLKO.1-puro vector were purchased (Transheep Bio). Transfection of these plasmids was performed using the Lipofectamine 3000 reagent (Invitrogen) according to the manufacturer’s instructions. Cells (2 × 10^5^) were seeded and infected by retrovirus generated by pMSCV-puro-cDNAs or pLKO.1-puro-shRNAs for 3 days. The stable cell lines were selected with 0.5 μg/ml puromycin for 7 days. The sequences of primers are provided in the Additional file 2: Table S5.

### 3-(4,5-Dimethyl-2-thiazolyl)-2,5-diphenyl-2H-tetrazolium bromide assay

Cells (0.2 × 10^4^ per well) were seeded in 96-well plates. At each time point, the cells were stained with 100 μl sterile 3-(4,5-Dimethyl-2-thiazolyl)-2,5-diphenyl- 2H-tetrazolium bromide (MTT) dye (0.5 mg/ml; Sigma) for 4 h at 37C, followed by removal of the culture medium and addition of 150 μl of dimethyl sulphoxide (Sigma). The absorbance was measured at 570 nm, with 655 nm as the reference wavelength. All experiments were performed in triplicate.

### Colony formation

Cells were plated on 6 well-plates (0.5 × 10^3^ cells per plate) and cultured for 10 days. The colonies were stained with 1% crystal violet for 30 s after fixation with 10% formaldehyde for 5 min.

### Bromodeoxyuridine (BrdU) incorporation assay

The bladder cancer cells were grown on cover slips. BrdU was added to the cells and incubated for 2 h. The cells were then fixed and stained with anti-BrdU antibody (Upstate, Temecula, CA, USA) according to the manufacturer’s instructions. The percentage of BrdU-positive cells in five random low-power fields was counted and reported as the mean ± SD.

### Luciferase activity assay

CCNE1 promoter from − 1750 to + 350 and CCNE2 promoter from − 2450 to + 350 were amplified by PCR, and then cloned into the pGL3 plasmid using the Sac II and Xho1 restriction enzymes. Primers for promoter amplification were: CCNE1-promoter-F: gccCCGCGGcctgttactggtgattcctaacg; R: gccCTCGAGgtgtcccctccacccca; CCNE2-promoter-F: gccAGATCTgaaaggggagactgggctg; R: gccGTCGACaaaaaaaggcacagaataaagaaat.

Luciferase assays were performed in stable cell lines with MNX1 overexpression or knockdown. Briefly, 3 × 10^4^ stably transfected cells were cultured in triplicate in 48-well plates for 24 h. Then, 100 ng luciferase reporter plasmids or the control-luciferase plasmid, plus 1 ng pRL-TK Renilla plasmid (Promega), were transiently transfected into the indicated stable cell lines using the Lipofectamine 3000 reagent (Invitrogen), according to the manufacturer’s recommendations. Luciferase and Renilla signals were measured 24 h after transfection, using the Dual Luciferase Reporter Assay Kit (Promega). Primers of the promoters were presented in the Additional file 2: Table S5.

### Chromatin immunoprecipitation (ChIP)

Cells (4 × 10^6^) in a 100 mm culture dish were treated with 1% final concentration of formaldehyde to cross-link proteins to DNA, and the reaction was stopped by addition of glycine. The cell lysates were sonicated to shear DNA to sizes of 300–1000 bp. Equal aliquots of chromatin supernatants were incubated with 1 μg of anti-MNX1, or anti-immunoglobulinG antibodies (Millipore, Billerica, MA, USA) overnight at 4 °C with rotation. After reverse cross-link of protein/DNA complexes to free DNA, PCR was performed. Specific primers for ChIP were presented in the Additional file 2: Table S6.

### Xenograft tumor model and tissue staining

Male BALB/c-nu mice (5–6 weeks old, 18–20 g) were purchased from the Slac-Jingda Animal Laboratory (Hunan, China), and housed in barrier facilities on a 12-h light/dark cycle. The Institutional Animal Care and Use Committee of Sun Yat-sen University approved all experimental procedures. The mice were randomly assigned to groups (*n* = 8 per group) and their dorsal flanks were subcutaneously injected with 1 × 10^6^ T24 cells. After 7 days, tumor formation kinetics were estimated by measuring tumor size at 3-day intervals. Tumor volume was calculated using the eq. (L*W^2^)/2. The animals were euthanized on day 42, and the tumors were excised, weighed, and paraffin-embedded. Serial 6.0-μm sections were obtained and stained with anti–Ki-67 (Dako, Glostrup, Denmark) and anti-BrdU.

### Statistical analysis

Statistical analyses were performed using the SPSS version 19.0 (SPSS Inc.) statistical software package. The log-rank test, χ^2^ test, Spearman rank correlation test, and Student *t*-test (two-tailed) were used. Multivariate statistical analysis was performed using a Cox regression model. Data are the mean ± SD. *P* < 0.05 was considered statistically significant.

## Results

### MNX1 was markedly upregulated in bladder cancer

**Fig. 1:**
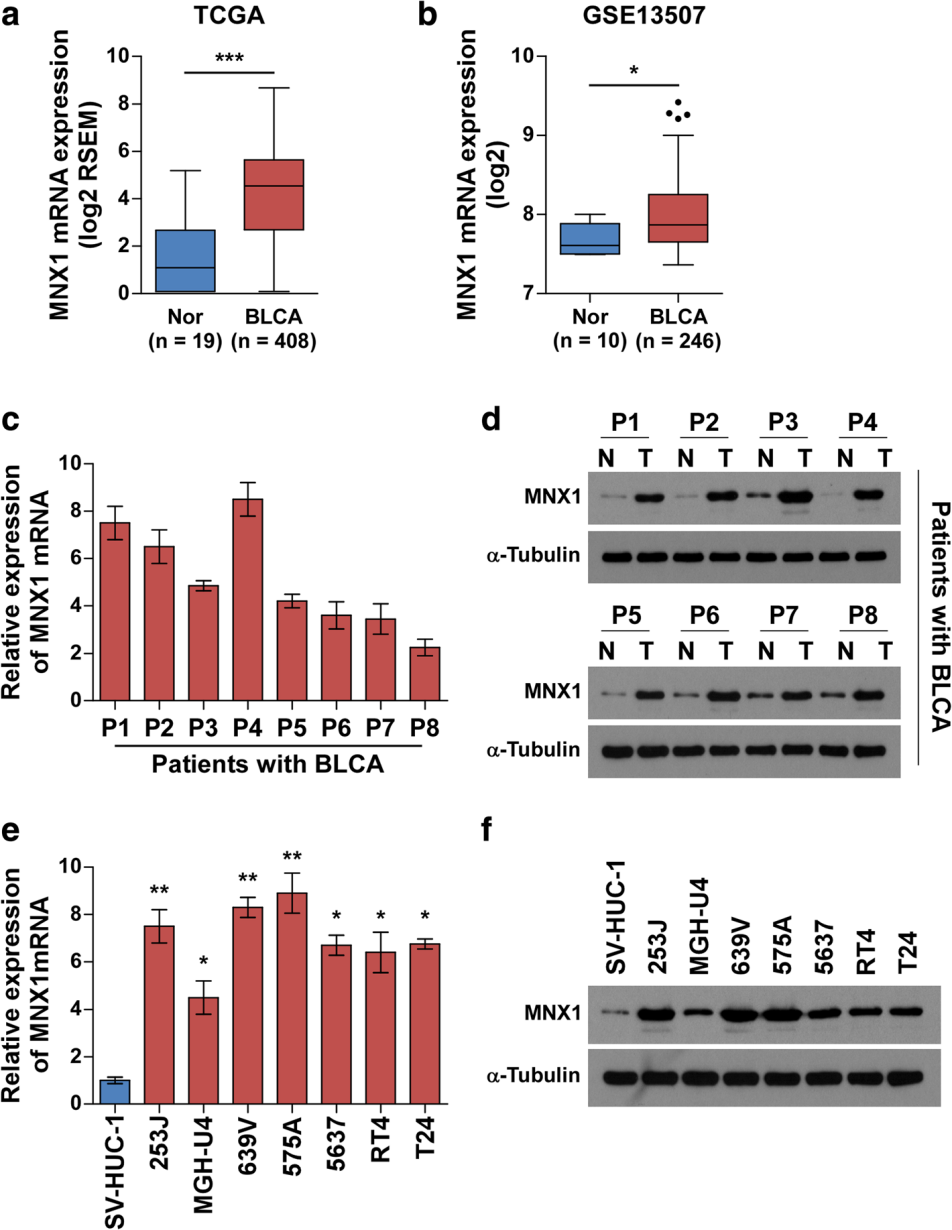
MNX1 is upregulated in bladder cancer tissues and cell lines. **a** *MNX1* mRNA expression levels in TCGA bladder cancer dataset including 19 normal bladder (Nor) and 408 bladder cancer (BLCA) samples. **b** *MNX1* mRNA levels were significantly upregulated in bladder cancer compared to normal bladder tissues by analyzing the GEO dataset (GSE13507), including 10 normal bladder (Nor) and 246 primary bladder cancer (BLCA) tissues. **c** and **d** Real-time PCR and western blotting of MNX1 mRNA (**c**) and protein (**d**) expression levels, respectively, in bladder cancer tissues (BLCA, T) versus the adjacent noncancerous tissues (N) from eight patients. **e** and **f** Real-time PCR and western blotting of MNX1 mRNA (**e**) and protein (**f**) expression levels, respectively, in seven human bladder cancer cell lines compared to the immortal SV-HUC-1 bladder cell line. Expression levels were normalized to glyceraldehyde-3-phosphate dehydrogenase (GAPDH) or α-tubulin. Bars represent the mean ± SD of three independent experiments. *p < 0.05; **p < 0.01; ***p < 0.001

To investigate the spectrum of MNX1 expression in bladder cancer, we first analyzed MNX1 expression in publicly available human bladder cancer datasets from The Cancer Genome Atlas (TCGA) [24] and Gene Expression Omnibus (GEO) [25]. MNX1 mRNA levels were obviously upregulated in bladder cancer samples compared with normal bladder tissues (Fig. 1a, b). We then detected MNX1 expression in bladder cancer cell lines and tissues. Western blotting and real-time PCR showed that MNX1 mRNA and protein expression, respectively, were markedly upregulated in all bladder cancer cell lines compared to primary normal urethral epithelial cells (Fig. 1c, d). Consistently, MNX1 expression was significantly higher in eight human bladder cancer tissues than in the paired adjacent non-tumor tissues (Fig. 1e, f). These findings demonstrate that MNX1 is upregulated in bladder cancer.

**Fig. 2:**
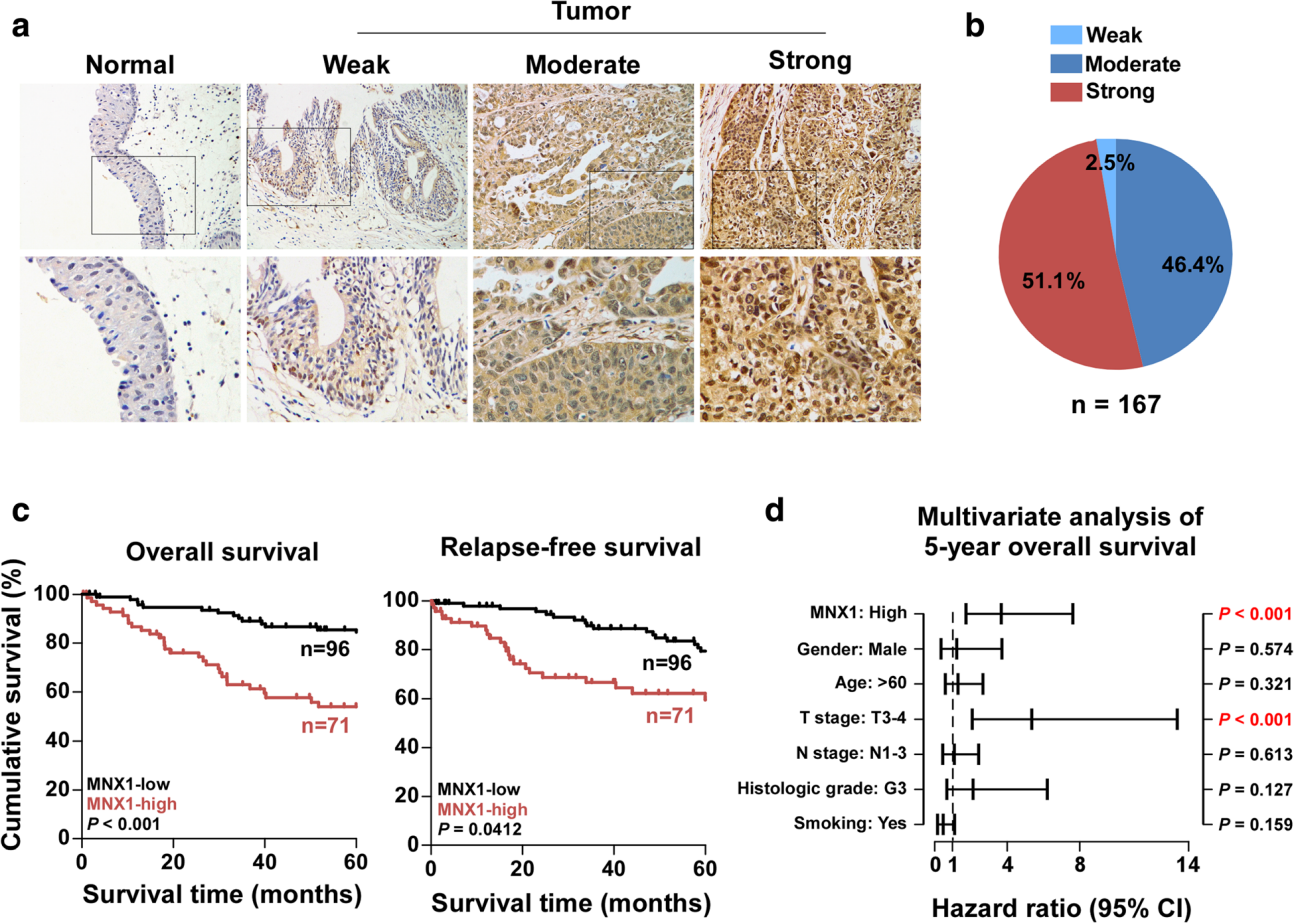
MNX1 upregulation is associated with poor prognosis in bladder cancer. **a** Representative images of MNX1 staining in normal and tumor tissues. Staining in the tumor group was scored as weak, moderate, or strong. Bottom row shows magnified inset area. **b** Distribution of MNX1 staining in bladder cancer specimens. **c** Kaplan–Meier 5-year overall survival (left) and relapse-free survival (right) curves for patients with bladder cancer stratified by low and high MNX1 expression (*n* = 167, log-rank test). **d** Kaplan–Meier 5-year overall survival (left) and relapse-free survival (right) curves for TCGA bladder cancer dataset stratified by low and high MNX1 expression (n = 167, log-rank test). 95% CI, 95% confidence interval; HR, hazard ratio

To evaluate the relationship between MNX1 expression and the clinicopathological features of bladder cancer, we analyzed 167 human bladder cancer tissue samples by IHC. Similarly, IHC indicated that MNX1 was greatly overexpressed in bladder cancer samples (Fig. 2a, b). The potential correlation between the expression status of MNX1 and clinical pathologic parameters of patients with bladder cancer was further analyzed (Additional file 1: Table S2). Furthermore, Kaplan–Meier survival curves and log-rank testing showed that patients with high MNX1 expression had shorter 5-year overall survival and relapse-free survival than those with low MNX1 expression (Fig. 2c). In addition, univariate Cox regression analysis indicated that the MNX1 expression and T stage status were each recognized as independent prognostic factors for the 5-year overall survival in bladder cancer (Fig. 2d and Additional file 1: Table S3). Taken together, these results show that MNX1 is upregulated in bladder cancer cell lines and tissues, and that it might lead to poor clinical outcome.

### MNX1 overexpression accelerated bladder cancer cell proliferation

**Fig. 3:**
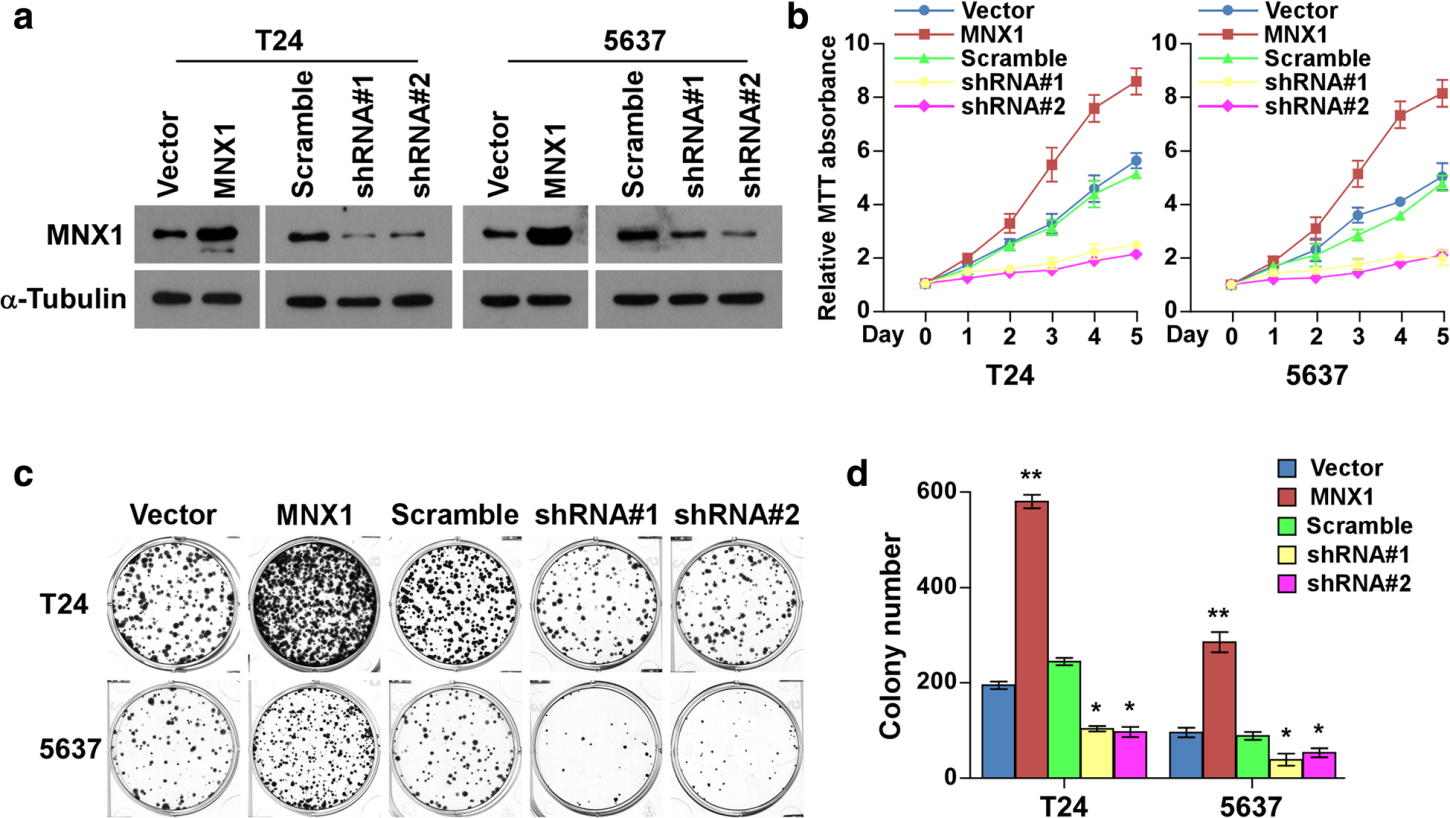
MNX1 overexpression accelerates bladder cancer cell proliferation. **a** MNX1 was stably overexpressed and silenced in T24 and 5637 cells. MNX1 expression was verified by western blotting. α-Tubulin was used as the loading control. **b** MTT assay assessment of cell viability. **c** and **d** Representative images (left) and quantification (right) of colony formation assay. Bars represent the mean ± SD of three independent experiments. *p < 0.05; **p < 0.01. shRNA, short hairpin RNA

To further investigate the effect of MNX1 in bladder cancer progression, we first established MNX1 stable overexpression and knockdown in T24 and 5637 cell lines (Fig. 3a). Strikingly, we found that MNX1 overexpression enhanced bladder cancer cell proliferation. The tetrazolium (MTT) assay showed that MNX1 upregulation increased the proliferation rate of bladder cancer cells; MNX1 depletion reduced it (Fig. 3b). The subsequent colony formation assay showed that MNX1 promoted bladder cancer cell colony numbers significantly, while silencing MNX1 had the opposite effects, and the colony numbers were counted and shown in histogram (Fig. 3c and d). These data suggest that MNX1 plays an important role in bladder cancer cell proliferation.

### Upregulating MNX1 drove G1–S transition in bladder cancer cells

**Fig. 4:**
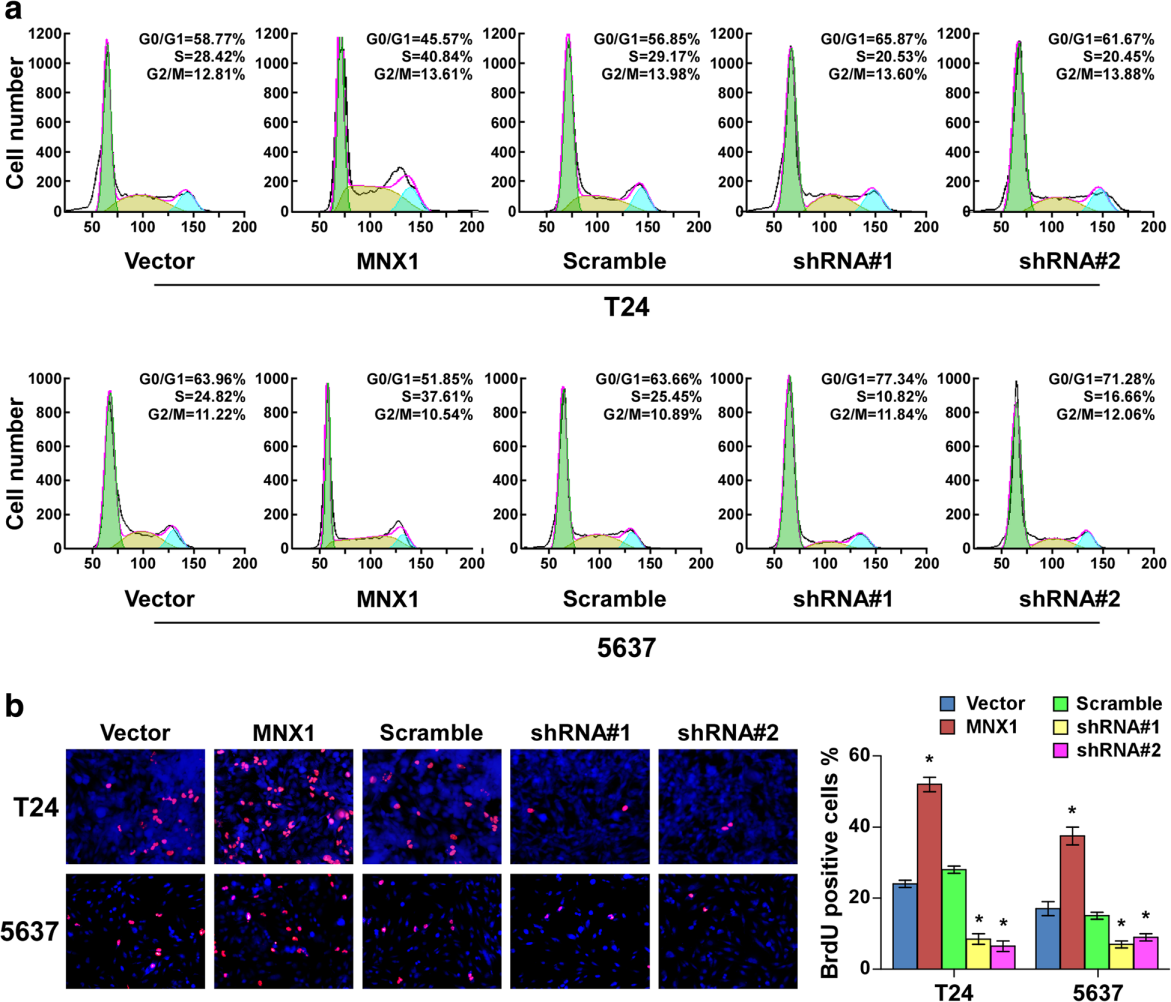
MNX1 upregulation drives G1–S transition in bladder cancer cell lines. **a** Flow cytometric analysis of bladder cancer cells with altered MNX1 expression. **b** Representative micrographs (left) and quantification (right) of BrdU-incorporating cells in MNX1-overexpressing or -silenced cells. *p < 0.05. shRNA, short hairpin RNA

To further evaluate whether this MNX1-induced promotion of cell proliferation was due to the inhibition of cell cycle arrest, we examined the effect of MNX1 on cell cycle of bladder cancer cells. Cell lines stably overexpressing MNX1 had significantly increased proportions of cells in the S phase, but reduced proportions of cells in the G1 phase. In contrast, silencing MNX1 had the opposite effects (Fig. 4a). The BrdU incorporation assay showed that the percentages of BrdU-incorporating cells were significantly enhanced in MNX1-overexpressing cells and were reduced in MNX1-silenced cells (Fig. 4b). These results indicate that MNX1 promotes cell cycle progression of bladder cancer cells, confirming that MNX1 promotes bladder cancer cell proliferation.

### MNX1 promoted bladder cancer cell tumorigenicity in vivo

**Fig. 5:**
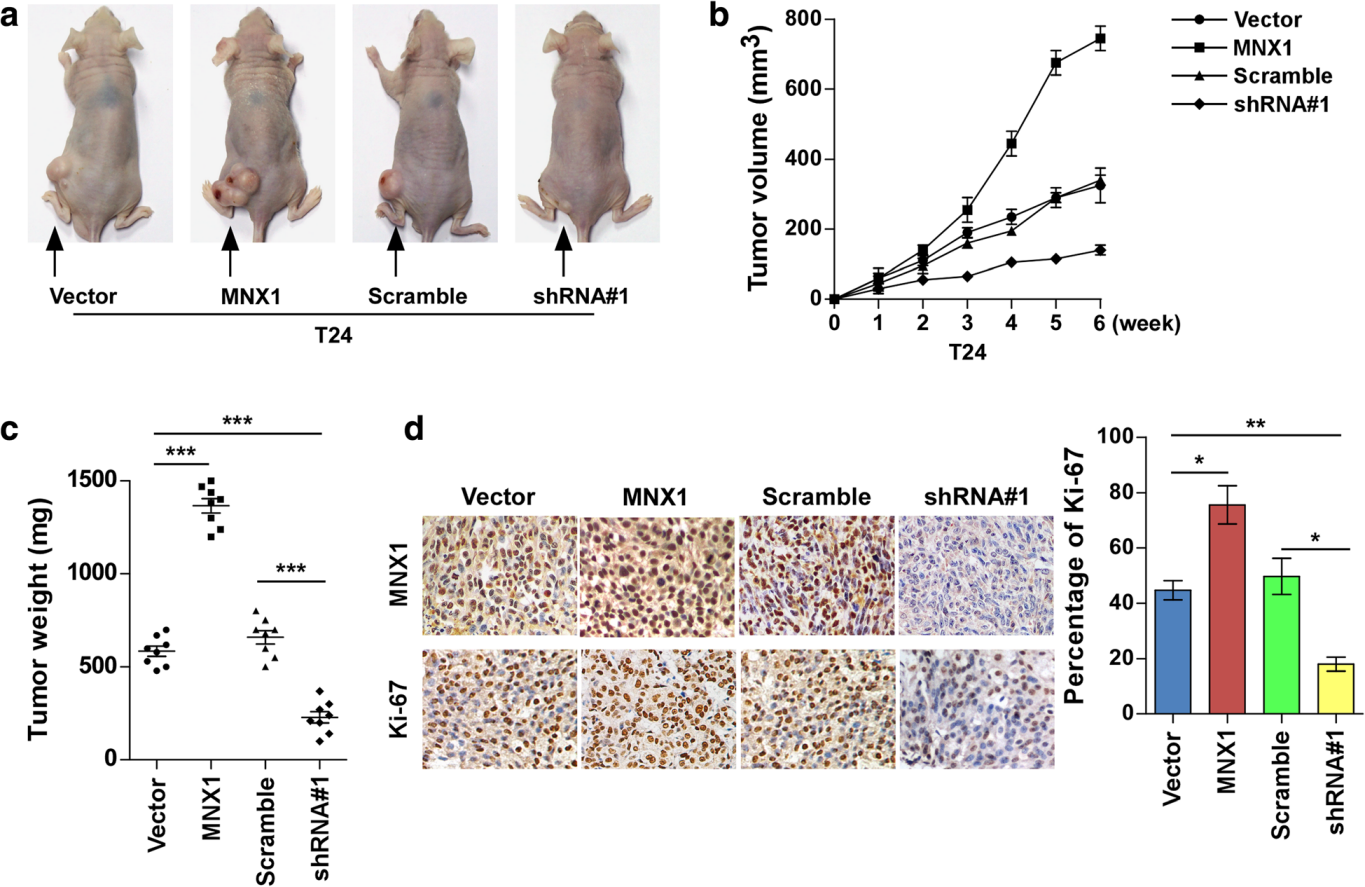
MNX1 promotes bladder cancer cell tumorigenicity in vivo. **a** Images show one representative mouse from each group. **b** Tumor volumes were calculated every week. **c** The primary tumors were excised and weighed at 6 weeks after transplantation. **d** IHC staining and histogram of Ki-67 in xenografts. *p < 0.05, ***p < 0.001. shRNA, short hairpin RNA

To further explore the effects of MNX1 in bladder cancer tumorigenesis in vivo, we established MNX1 stable overexpression and knockdown in T24 cells (Fig. 5a). MNX1-overexpressing, MNX1 knockdown, or vector cells were subcutaneously injected into BALB/c-nu mice, and the tumor growth was measured. The MNX1-overexpressing group had prominently increased tumor growth, whereas it was obviously suppressed in the MNX1 knockdown group, both as compared with the vector group (Fig. 5b, c). Moreover, tumors from the MNX1 upregulation group exhibited high expression of the proliferation marker Ki-67 compared to the vector group. In contrast, MNX1 downregulation was associated with low Ki-67 expression (Fig. 5d). These results indicate that MNX1 promotes bladder cancer cell growth in vivo.

### MNX1 directly upregulated CCNE1 and CCNE2 promoter activity in bladder cancer cells

**Fig. 6:**
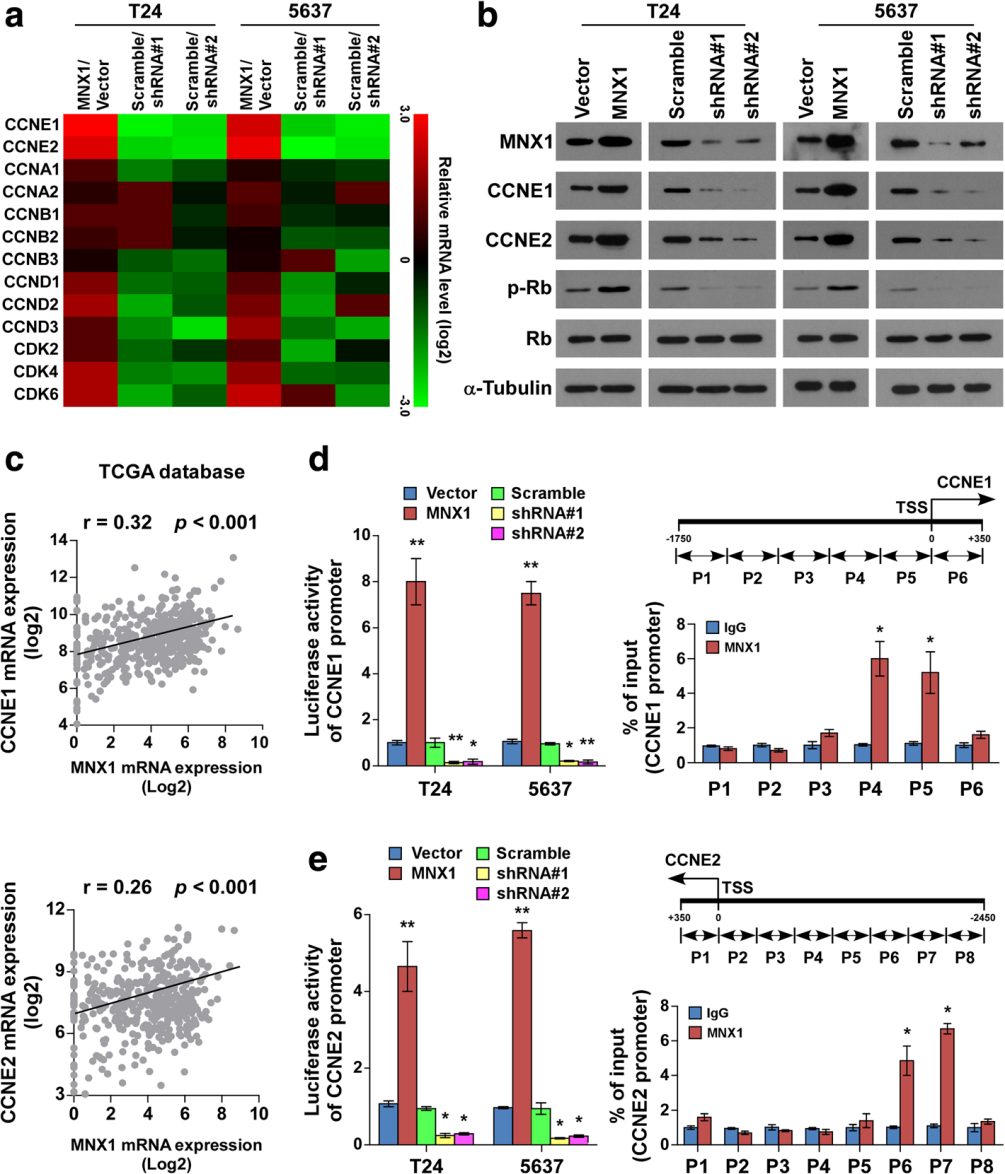
MNX1 directly upregulates *CCNE1* and *CCNE2* promoter activity in bladder cancer cells. **a** Real-time PCR of cell cycle–related gene mRNA expression. Gene expression levels were normalized to *GAPDH*. Pseudo-colors represent the intensity scale of MNX1 versus vector or MNX1 shRNA#1/2 versus Scramble, generated by log2 transformation. **b** Western blotting of CCNE1, CCNE2, phosphorylated Rb (p-Rb), and total Rb protein expression; α-tubulin was used as the loading control. **c** The scatter diagram of CCNE1 and CCNE2 from cBioportal program. **d** and **e** Left: Luciferase activity assays of T24 and 5637 cells showed transactivation and repression of the *CCNE1* (**d**) and *CCNE2* (**e**) promoters by MNX1 overexpression and MNX1 knockdown, respectively. Right: Schematic illustration of ChIP PCR fragments for the indicated nucleotide regions of the *CCNE1* (**d**) and *CCNE2* (**e**) promoters (top). The ChIP enrichment assay confirmed that MNX1 binds to the P4 and P5 promoter of *CCNE1* (**d**) and the P6 and P7 promoters of *CCNE2* (**e**); immunoglobulin G (IgG) was used as the negative control. The results from three independent experiments were evaluated. *p < 0.05; **p < 0.01. shRNA, short hairpin RNA

As MNX1 is involved in the cell cycle regulation of bladder cancer cells, we examined the expression of cell cycle regulators. Real-time PCR and western blotting revealed that multiple cell cycle regulators, especially CCNE1 and CCNE2, were robustly increased in MNX1-overexpressing cells, but were reduced in MNX1-silenced cells compared to the control cells (Fig. 6a, b). Moreover, phosphorylation of Rb, the downstream target protein of the CCNE–CDK2 complex, was induced in MNX1-overexpressing cells, but was suppressed in the MNX1-silenced cells (Fig. 6b). Consistently, using the cBioportal program (http://www.cbioportal.org), we found that most of these regulators were positively correlated with MNX1 expression (Additional file 3: Figure S1). Intriguingly, among these cyclin genes, CCNE1 and CCNE2 showed the highest correlation with MNX1 (*r* = 0.32 and 0.26), suggesting that MNX1 might play a role in CCNE1/2 transcription (Fig. 6c). As expected, luciferase reporter assays revealed that MNX1 overexpression activated the luciferase activity of CCNE1 and CCNE2 promoters in the bladder cancer cells, whereas MNX1 downregulation attenuated it (Fig. 6d, e). We then examined whether MNX1 upregulates CCNE1 and CCNE2 transcriptionally using chromatin immunoprecipitation (ChIP). The ChIP showed that MNX1 can bind to different regions within the CCNE1 and CCNE2 promoters (Fig. 6d, e). The above data indicate that MNX1 regulates CCNE1 and CCNE2 by directly targeting their promoter elements.

## Discussion

MNX1 protein has been proven to be an important regulator of many processes relevant to cancer. For example, MNX1 is involved in a recurrent translocation specifically found in infant AML, in which the MNX1 gene is frequently fused to the ETV6 gene on chromosome 1 [14]. It was also recently determined that MNX1 plays a significant role in cell proliferation in human insulinomas [26]. Moreover, MNX1 is associated with normal cells malignant transformation depending on its mutation causes congenital malformation---Currarino syndrome, suggesting that MNX1 may be a potential driver in tumorigenesis. However, the clinical importance and biological role of MNX1 in bladder cancer remain largely unknown. Herein, we found that MNX1 was robustly upregulated in bladder cancer, and established a vital role for MNX1 as a tumor-promoting factor of bladder cancer proliferation and tumorigenicity. Strikingly, IHC could detect MNX1 at all T classifications in the bladder cancer specimens and it correlated with poor outcome. Therefore, our results suggest that MNX1 may be an oncogene and might represent a novel prognostic biomarker in bladder cancer.

Patients with early-stage or localized bladder cancer can be managed by surgical resection, while those with advanced bladder cancer are usually treated with radiotherapy or chemotherapy. Although efficient treatment is administered, the therapeutic outcomes remain unsatisfactory [27]. Our data reveal that ectopic expression of MNX1 promoted the formation of subcutaneous tumors in vivo, while silencing MNX1 inhibited it, which indicates MNX1 is an important factor in bladder cancer cell tumorigenicity. Our findings suggest that MNX1 is a potential therapeutic target against bladder cancer.

Generally, dysregulation of CCNE1/2 activity is present in various cancers [28–31], resulting in disrupted G1–S transition and uncontrolled cell proliferation. In the present work, MNX1 overexpression induced the expression of multiple cell cycle regulators and upregulated CCNE1 and CCNE2 by directly targeting their promoter elements, leading to G1–S transition and a high cell proliferation rate. Involvement of the CCNE–CDK2 complex is well-established in cell cycle regulation, playing an important role in tumor development [32, 33]. The E2F transcription factors strongly activate CCNE1 and CCNE2, and the CCNE–CDK2 complex phosphorylates and inactivates Rb, while the phosphorylated Rb releases E2F transcription factors, thereby promoting cell cycle progression from G1 to S phase [34–37]. Herein, we investigated that MNX1 upregulates CCNE1 and CCNE2 expression to induce proliferation and tumorigenicity in bladder cancer by targeting their promoters. However, we have not figured out the specific sequence of CCNE1 and CCNE2 that MNX1 directly binds to. The detail mechanisms remain to be clarified in future. These observations reveal a new molecular mechanism of MNX1 in bladder cancer.

## Conclusions

In summary, our study reveals that MNX1 upregulation plays an important role in bladder cancer progression and that it is a critical cell cycle promoter that upregulates CCNE1 and CCNE2 directly. However, more MNX1 mechanisms and functions in bladder cancer require further exploration. These findings will not only advance our understanding of the mechanism underlying cell cycle regulation and tumorigenicity, but also establish MNX1 as a key regulator of bladder cancer progression and a valuable prognostic marker, and may also facilitate the development of new therapeutic strategies against bladder cancer.

## Additional files

Additional file 1: **Table S1.** Clinicopathological characteristics of 167 patient samples. **Table S2.** Correlation between MNX1 and clinicopathological characteristics of bladder cancer patients. **Table S3.** Univariate and multivariate analysis of factors associated with overall survival in 167 bladder cancer patients. (DOCX 24 kb)

Additional file 2: **Table S4.** Primers for real-time PCR analysis. **Table S5.** Primers for plasmid constructs. **Table S6.** Primers for ChIP. (DOCX 18 kb)

Additional file 3: **Figure S1.** Pearson score of the indicated cell cycle regulators from cbioportal. (DOCX 83 kb)

## Abbreviations

BrdU: Bromodeoxyuridine
CCNE1/2: Cyclin E1/2
CDK: Cyclin-dependent kinase
ChIP: Chromatin immunoprecipitation
GEO: Gene Expression Omnibus
IHC: Imunohistochemistry
MNX1: Motor neuron and pancreas homeobox 1
Rb: Retinoblastoma
TCGA: The Cancer Genome Atlas
