# Supplementary material for: Motor neuron and pancreas homeobox 1/HLXB9 promotes sustained proliferation in bladder cancer by upregulating CCNE1/2
Source: J Exp Clin Cancer Res. 2018 Jul 16;37:154. doi: 10.1186/s13046-018-0829-9 (PMC6048799; doi:10.1186/s13046-018-0829-9)
Supplement: Supplementary file 1 — Table S1. Clinicopathological characteristics of 167 patient samples. Table S2. Correlation between MNX1 and clinicopathological characteristics of bladder cancer patients. Table S3. Univariate and multivariate analysis of factors associated with overall survival in 167 bladder cancer patients. (DOCX 24 kb) [file 13046_2018_829_MOESM1_ESM.docx]

**Additional file 1:** **Table S1-3**

**Table S1 Clinicopathological characteristics of 167 patient samples**

| Parameters | Number of cases (%) |
| --- | --- |
| **Gender** |  |
| Female | 20 (12.0) |
| Male | 147 (88.0) |
| **Age (years)** |  |
| ≤ 60 | 84 (50.3) |
| > 60 | 83 (49.7) |
| **T classification** |  |
| T_1_ | 15 (9.0) |
| T_2_ | 65 (38.9) |
| T_3_ | 43 (25.7) |
| T_4_  T_x_ | 39 (23.4)  5 (3.0) |
| **N classification** |  |
| N_0_ | 93 (55.7) |
| N_1_ | 14 (8.4) |
| N_2_ | 19 (11.4) |
| N_3_  N_x_ | 7 (4.2)  34 (20.3) |
| **M classification** |  |
| Negative | 71 (42.5) |
| Positive  x | 7 (4.2)  89 (53.3) |
| **Histologic grade** |  |
| Low grade | 18 (10.8) |
| High grade | 142 (85.0) |
| x | 7 (4.2) |
| **Smoking** |  |
| No | 65 (38.9) |
| Yes  x | 84 (50.3)  18 (10.8) |
| **Vital status** |  |
| Dead | 57 (34.1) |
| Alive | 110 (65.9) |
| **Relapse status** |  |
| Negative | 96 (57.5) |
| Positive | 71 (42.5) |
| **MNX1 expression** |  |
| Low | 96 (57.5) |
| High | 71 (42.5) |

**Table S2 Correlation between MNX1 and clinicopathological characteristics of bladder cancer patients**

|  | | | |
| --- | --- | --- | --- |
|  | **MNX1 expression** | |  |
| Characteristics | Low,  no. cases | High,  no. cases | *P* values |
| **Gender** |  |  |  |
| Female | 13 | 7 | 0.469 |
| Male | 83 | 64 |  |
| **Age (years)** |  |  |  |
| ≤ 60 | 56 | 28 | 0.016 |
| > 60 | 40 | 43 |  |
| **T** **classification** |  |  |  |
| T_1 –_ T_2_ | 47 | 33 | 0.853 |
| T_3_ – T_4_ | 47 | 35 |  |
| **N classification** |  |  |  |
| N_0_ | 53 | 40 | 0.956 |
| N_1_ – N_3_ | 23 | 17 |  |
| **M classification** |  |  |  |
| Negative | 43 | 28 | 0.860 |
| Positive | 4 | 3 |  |
| **Histologic grade** |  |  |  |
| Low grade | 7 | 11 | 0.102 |
| High grade | 84 | 58 |  |
| **Smoking** |  |  |  |
| No | 39 | 26 | 0.837 |
| Yes | 49 | 35 |  |
| **Vital status** |  |  |  |
| Dead | 24 | 33 | 0.004 |
| Alive | 72 | 38 |  |
| **Relapse status** |  |  |  |
| Negative | 54 | 42 | 0.707 |
| Positive | 42 | 29 |  |

**Table S3 Univariate and multivariate analysis of factors associated with overall survival in 167 bladder cancer patients.**

| Characteristics | Univariate analysis | | | Multivariate analysis | |
| --- | --- | --- | --- | --- | --- |
|  | HR (95% CI) | *P* values | HR (95% CI) | | *P* values |
| **MNX1 expression**  (high) | 2.798 (1.637-4.780) | < 0.001 | 3.794 (1.858-7.744) | | < 0.001 |
| **Gender**  (male) | 1.069 (0.458-2.494) | 0.878 | 1.350 (0.475-3.838) | | 0574 |
| **Age**  (> 60) | 1.658 (0.987-2.875) | 0.056 | 1.411 (0.715-2.787) | | 0.321 |
| **T classification**  (T_3_ – T_4_) | 3.880 (2.129-7.069) | < 0.001 | 5.477 (2.197-13.500) | | < 0.001 |
| **N classification**  (N_1_ – N_3_) | 1.736 (0.940-3.207) | 0.078 | 1.212 (0.576-2.551) | | 0.613 |
| **Histologic grade** (High grade) | 1.021 (0.436-2.394) | 0.961 | 2.244 (0.795-6.332) | | 0.127 |
| **Smoking**  (yes) | 0.851 (0.485-1.491) | 0.572 | 0.593 (0.287-1.227) | | 0.159 |

HR, hazard ratio; CI, confidence interval.
