# Supplementary material for: Motor neuron and pancreas homeobox 1/HLXB9 promotes sustained proliferation in bladder cancer by upregulating CCNE1/2
Source: J Exp Clin Cancer Res. 2018 Jul 16;37:154. doi: 10.1186/s13046-018-0829-9 (PMC6048799; doi:10.1186/s13046-018-0829-9)
Supplement: Supplementary file 2 — Table S4. Primers for real-time PCR analysis. Table S5. Primers for plasmid constructs. Table S6. Primers for ChIP. (DOCX 18 kb) [file 13046_2018_829_MOESM2_ESM.docx]

**Additional file 2:** **Table S4-6**

**Table S4 Primers for real-time PCR analysis**

| Gene |  | Sequence(5'-3') |
| --- | --- | --- |
| MNX1 | forward | CTCCTACTCGTACCCGCAG |
|  | reverse | TTGAAGTCGGGCATCTTAGGC |
| CCNE1 | forward | GCCAGCCTTGGGACAATAATG |
|  | reverse | CTTGCACGTTGAGTTTGGGT |
| CCNE2 | forward | TCAAGACGAAGTAGCCGTTTAC |
|  | reverse | TGACATCCTGGGTAGTTTTCCTC |
| GAPDH | forward | AAGGTGAAGGTCGGAGTCAA |
|  | reverse | AATGAAGGGGTCATTGATGG |

**Table S5 Primers for plasmid constructs**

| Constructs |  | Sequence(5'-3') |  |  |
| --- | --- | --- | --- | --- |
| pMSCV-MNX1 | forward | GCCAGATCTGCCACCATGGAAAAATCCAAAAATTT  CCGCATCGAC | |  |
|  | reverse | GCCGAATTCCTACTGGGGCGCGGGCTGGT | | |
| pGL3-CCNE1 -promoter | forward | gccCCGCGGcctgttactggtgattcctaacg | | |
|  | reverse | gccCTCGAGgtgtcccctccacccca | | |
| pGL3-CCNE2-promoter | forward | gccAGATCTgaaaggggagactgggctg | | |
|  | reverse | gccGTCGACaaaaaaaggcacagaataaagaaat | | |
|  |  |  | | |

**Table S6 Primers for ChIP**

| **Gene (promoter)** |  | **Sequence(5'-3')** |
| --- | --- | --- |
| CCNE1 P1 | forward | CCTGTTACTGGTGATTCCTAACG |
|  | reverse | GAGACACTTAAATTGTTTCTGAATGA |
| CCNE1 P2 | forward | TGTCTCCCTTGGTCCAGGCA |
|  | reverse | TCAAATCCACACTCCGTGTCCTAG |
| CCNE1 P3 | forward | TGTGGAGCCTGTAGCCTAGGA |
|  | reverse | GTCAGTGCATTGGGTCGTTC |
| CCNE1 P4 | forward | CAATGCACTGACGGATGAA |
|  | reverse | CCCCGCCCCTGATTCC |
| CCNE1 P5 | forward | CTTTTTGCCGCTCCAGC |
|  | reverse | GCTCAGAGCGGGACATTTA |
| CCNE1 P6 | forward | ATTTTAAATGTCCCGCTCTGAG |
|  | reverse | GTGTCCCCTCCACCCCA |
| CCNE2 P1 | forward | GAAAGGGGAGACTGGGCTG |
|  | reverse | CGAGCGGTAGCTGGTCTGG |
| CCNE2 P2 | forward | CATGCGCCTCAGACTGACAC |
|  | reverse | CCGAGTCGTGTCCCCCT |
| CCNE2 P3 | forward | GGGACACGACTCGGGC |
|  | reverse | AAAGCAGGGTTGATACATACCT |
| CCNE2 P4 | forward | AGGTATGTATCAACCCTGCTTTCC |
|  | reverse | TGCACGTGGTGGCGATC |
| CCNE2 P5 | forward | GGAAGAAAGATCGCCACCAC |
|  | reverse | CCGAGACGCCTTCGCAC |
| CCNE2 P6 | forward | GGGAGCCCTTCCCGAGAT |
|  | reverse | GAGAGCCATCCTAAGCGTTAGAA |
| CCNE2 P7 | forward | TGCAGTCACTCAAAACGCAGT |
|  | reverse | CTATGATTGCATTCTGTTCACATAATA |
| CCNE2 P8 | forward | TTGTGTGGAAAGGAATAGAGTGAAT |
|  | reverse | AAAAAAAGGCACAGAATAAAGAAAT |
