# Supplementary material for: Motor neuron and pancreas homeobox 1/HLXB9 promotes sustained proliferation in bladder cancer by upregulating CCNE1/2
Source: J Exp Clin Cancer Res. 2018 Jul 16;37:154. doi: 10.1186/s13046-018-0829-9 (PMC6048799; doi:10.1186/s13046-018-0829-9)
Supplement: Supplementary file 3 — Figure S1. Pearson score of the indicated cell cycle regulators from cbioportal. (DOCX 83 kb) [file 13046_2018_829_MOESM3_ESM.docx]

**Additional file 3: Figure S1** Pearson score of the indicated cell cycle regulators from cbioportal.

**
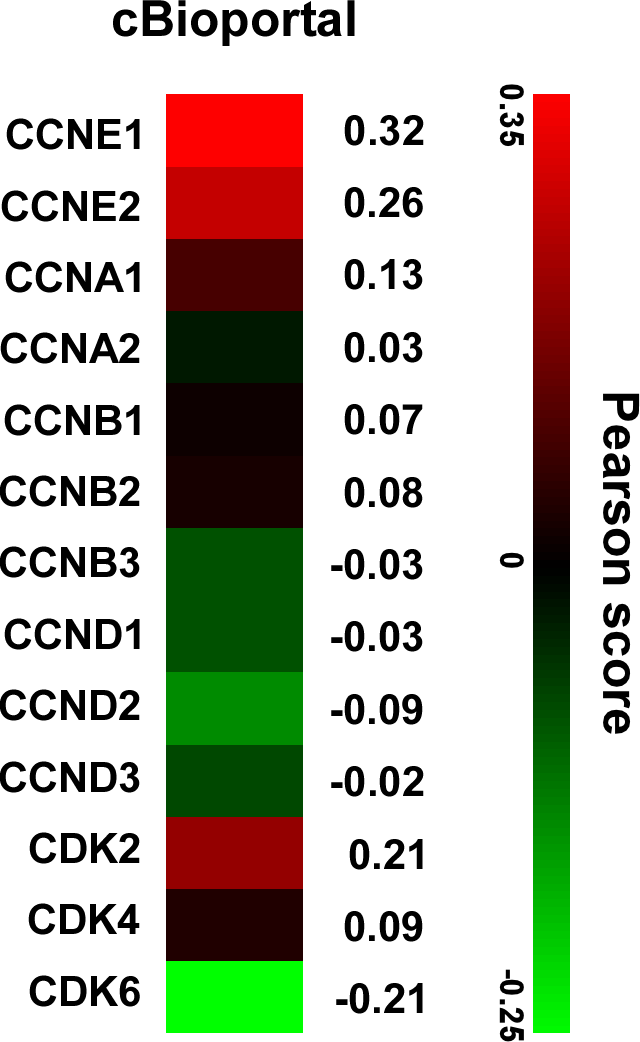
**
